# Factors Associated with Quit Intentions among Adult Smokers in South Korea: Findings from the 2020 ITC Korea Survey

**DOI:** 10.3390/ijerph191710839

**Published:** 2022-08-31

**Authors:** Minjung Han, Donghee Seo, Yeol Kim, Hong Gwan Seo, Sung-il Cho, Sungkyu Lee, Sujin Lim, Susan C. Kaai, Anne C. K. Quah, Mi Yan, Steve S. Xu, Geoffrey T. Fong

**Affiliations:** 1 Department of Family Medicine, Myongji Hospital, Goyang 10475, Korea; 2 Department of Family Medicine, National Cancer Center, Goyang 10408, Korea; 3 Department of Cancer Control, Graduate School of Cancer Science and Policy, National Cancer Center, Goyang 10408, Korea; 4 Graduate School of Public Health, Seoul National University, Seoul 08826, Korea; 5 Korea Center for Tobacco Control Research and Education, Seoul 04554, Korea; 6 National Tobacco Control Center, Korean Health Promotion Institute, Seoul 04933, Korea; 7 Department of Psychology, University of Waterloo, Waterloo, ON N2L 3G1, Canada; 8 School of Public Health Sciences, University of Waterloo, Waterloo, ON N2L 3G1, Canada; 9 Ontario Institute for Cancer Research, Toronto, ON M5G 0A3, Canada

**Keywords:** smoking, quit intentions, predictors, cessation, South Korea

## Abstract

**Background**: South Korea has made substantial progress on tobacco control, but cigarette smoking prevalence is still high. Previous studies were conducted before the use of nicotine vaping products (NVPs) or heated tobacco products (HTPs) became popular. Thus, whether the concurrent use of NVPs or HTPs affects quit intentions among Korean smokers remains a question that needs to be explored. This study aims to identify predictors of quit intentions among cigarette-only smokers and concurrent users of cigarettes and NVPs or HTPs. **Methods**: Data were from the 2020 International Tobacco Control Korea Survey. Included in the analysis were 3778 adult cigarette smokers: 1900 at-least-weekly exclusive smokers and 1878 at-least-weekly concurrent smokers and HTP or NVP users. Bivariate and multivariable logistic regression analyses were conducted. **Results**: Quit intentions were reported by 66.4% of respondents. Factors significantly associated with quit intentions included younger age, having a spouse/partner, lower nicotine dependence, reporting a past quit attempt, regretting starting smoking, believing that smoking had damaged health, worrying that smoking will damage future health, and perceiving health benefits of quitting. Current use of NVPs or HTPs was not significantly associated with quit intentions. **Conclusions**: This study contributes the following to current literature: intrinsic health-related beliefs were more important than societal norms in shaping quit intentions. These findings should be considered in shaping future smoking cessation policies, such as reinforcing education programs that emphasize the benefits of quitting for personal health reasons, lowering nicotine dependence, and encouraging multiple quit attempts and successful quitting.

## 1. Introduction

Tobacco use is a leading cause of preventable disease and death around the world. More than eight million deaths have been attributed to tobacco use each year [1]. According to the most recent report by the World Health Organization, approximately one billion people aged 15 and older, including 847 million men and 153 million women, currently smoke [1]. Additionally, 24 million children aged 13–15 years are estimated to smoke globally [1]. While the global smoking prevalence rate has slowly decreased, smoking remains a public health problem in many parts of the world, including the Republic of Korea (South Korea, hereafter) [2,3]. Despite attempts to reduce smoking rates across the world, smoking remains a major preventable risk factor for non-communicable diseases such as chronic obstructive pulmonary disease (COPD), cardiovascular diseases, and various cancers [2,4]. 

South Korea has a long history of tobacco control and has made substantial progress on tobacco control [5]. The National Health Promotion Act (NHPA) came into effect in 1995 to mitigate the negative effects of tobacco use [5]. The NHPA allowed the government to impose numerous smoking bans, expand nationwide smoking cessation services, increase smoke-free zones, and implement anti-tobacco campaigns [6]. In 2004 and 2015, the average retail price of cigarette packs was raised by 29% and 80%, respectively, because of the tobacco tax increase [6]. In 2005, nationwide government-sponsored smoking cessation clinics were established [7]; in the same year, the expansion of anti-smoking campaigns took place to include not only television advertisements but also other types of media, such as internet and poster media [6]. Since 2013, smoking has been prohibited in public indoor areas including restaurants, bars, and coffee shops [7]. In 2015, the National Insurance Cooperation began to cover the expenses for smoking cessation programs, rendering these health services nearly free of charge for smokers [7]. In 2016, a mandate was issued to include graphic health warnings on cigarette packaging [6]. In spite of all these efforts, 54,500 South Koreans die yearly from tobacco-related diseases; more than 59,000 children (10–14 years old) and 9,081,000 adults (15+ years) continue to use tobacco daily [7]. 

Adult smoking rates in South Korea have dropped drastically over the past few decades, from 75% of adult men in 1992 to 38.1% in 2017 [5,6]. However, in recent years, male smoking rates plateaued at 36.7%, 35.7%, and 34% in 2018, 2019, and 2020, respectively [8]. Female smoking rates have also followed a similar trend, i.e., 7.5%, 6.7%, and 6.6% in 2018, 2019, and 2020, respectively [8]. Coinciding with the plateau in smoking rates were dramatic changes in the tobacco landscape [5,6]. Following the initial success of IQOS in Japan, a heated tobacco product (HTP), Philip Morris International (PMI) was the first tobacco company to enter the South Korean market in May 2017 [5]. Immediately following the footsteps of PMI, Korea Tobacco and Ginseng Corporation (KT&G) and British American Tobacco (BAT) launched and marketed their own HTPs, lil and Glo, respectively, in 2018 [5]. In recent years, a new generation of nicotine vaping products (NVPs), such as Juul pods and lil vapor, also became popular among consumers. 

Smoking is a complex behavior that has been studied using several theoretical frameworks. According to the transtheoretical model of change, the process of smoking cessation involves five stages of behavior change: precontemplation, contemplation, preparation, action, and maintenance [9]. Having quit intentions is classified under the preparation stage and is a strong predictor of making future quit attempts and successful quitting [10]. Factors associated with having quit intentions vary across nations, reflecting the societal and cultural differences across countries. Predictors of quit intentions from previous studies included sociodemographics (sex, age, income, and education), history of quit attempts, duration of previous quit attempts, nicotine dependence, beliefs in quitting, benefits of quitting, self-efficacy, future health, opinion on smoking, quality of life, stress, chronic diseases, exposure to anti-smoking campaigns, smoking restrictions, and quit advice from health professionals [11,12,13,14,15,16,17,18,19,20,21,22]. 

Few studies assessing the factors associated with quit intentions have been conducted in South Korea (see Supplementary Table S1) [18,23,24,25,26,27,28]. Two of these studies [18,24], conducted by the International Tobacco Control Policy Evaluation (ITC) Project, reported that sociodemographic factors, smoking-related beliefs, smoking restrictions at home, tobacco-related knowledge, and nicotine dependence were associated with having a quit intention [18,24]. However, it has been over a decade since the last comprehensive study of predictors of quit intentions was conducted in Korea. Since then, stronger tobacco control policies have been implemented in the nation, as described previously. Reflecting changes to policy, predictors of quit intentions may have also evolved, necessitating an update of existing studies. Additionally, previous studies were conducted before the introduction of NVPs or HTPs, and thus there is a need to understand whether the concurrent use of these other tobacco/nicotine products may be affecting quit intentions in Korean smokers. The aim of this study was to investigate known factors from the literature that are associated with having quit intentions, including those that were not examined in previous studies, using data from the 2020 ITC Korea Survey.

## 2. Methods

Data were from Wave 1 of the third cohort ITC Korea (KRA1) Survey, which was a web survey conducted 18–28 June 2020 across 4794 adults (aged ≥19): at-least-weekly exclusive cigarette smokers, HTP users, and NVP users; at-least-weekly concurrent users of HTPs and cigarettes, NVPs and cigarettes, and NVPs and HTPs; at-least-weekly triple users; and non-users [5,29]. For exclusive cigarette smokers and non-users, quotas were implemented based on geographical regions, sex, and age group to obtain a final sample size proportional to stratum size based on South Korean smoking prevalence estimates [5,29]. The sample was recruited from Rakuten Insight’s proprietary online panel in South Korea. The response rate was 15.2% and the cooperation rate was 97.4% [5,29].

A detailed description of the ITC KRA1 sample and methods is reported elsewhere [5,29]. Study procedures and materials were reviewed and cleared by the Research Ethics Board, Office of Research Ethics at the University of Waterloo, Canada (REB#41512) and the Institutional Review Board of Korean Health Promotion Institute (#120160811107AN01-2004-HR-042-02). All participants provided consent prior to completing the survey.

### 2.1. Study Sample 

The study sample consisted of 3778 cigarette smokers: of these, 1900 were exclusive smokers and 1878 were concurrent smokers and NVP and/or HTP users. Of these 1878 concurrent smokers and HTP and/or NVP users, 1136 were concurrent HTP and cigarette users, 228 were concurrent NVP and cigarette users, and 514 were users of all three products. 

### 2.2. Measures 

All current smokers were asked if they were planning to quit smoking (dependent variable). Those reporting “within the next month”, “between 1 and 6 months”, or “sometime in the future, beyond 6 months” were classified as having quit intentions, while those who responded “not planning to quit” were classified as not having quit intentions. Sociodemographic variables were sex (male and female); age (19–29, 30–39, 40–59, 60+); education (low: no schooling/primary/middle school, moderate: high school/some university, and high: university degree/post-graduate); annual household income (low: lower than KRW10 million (~$7700 USD), moderate: between KRW10 million (~$7700 USD) and KRW60 million (~$46,200 USD), high: higher than KRW60 million (~$46,200USD), not stated: respondents refused to answer or answered “Don’t know”; marital status (married/common law, separated/divorced/widowed, and single); and tobacco use status (exclusive smoker and concurrent smoker).

Nicotine dependence was measured by using the Heaviness of Smoking Index (HSI), which was based on the sum of two categorical variables: number of cigarettes smoked per day (CPD; 0: 0–10, 1: 11–20, 2: 21–30, and 3: 31+) and time to first cigarette in minutes: 0: >60, 1: 31–60, 2: 6–30, 3: ≤5. Smokers also reported if they had ever tried to quit smoking cigarettes (recoded as: never tried to quit, no quit attempt in the past year, and one or more attempts in the past year).

Psychosocial beliefs about smoking were assessed by asking for rating of agreement with these two statements: “If you had to do it again, you would not have started smoking” (recoded as: strongly agree/agree, neutral, and disagree/strongly disagree), and “Smoking cigarettes is an important part of my life” (recoded as: strongly agree/agree, neutral, and disagree/strongly disagree).

Perceived risk was assessed by asking smokers four questions: (1) to what extent smoking lowered their quality of life (recorded as: not at all/just a little and a fair amount/a great deal), (2) to what extent smoking cigarettes had damaged their health (recoded as: not at all/just a little and a fair amount/a great deal), (3) how worried they were that smoking cigarettes would damage their health in the future (recorded as: not at all worried and a little/moderately/very much worried), and (4) if they continued to smoke, what were the chances that they would contract a smoking-related disease such as lung cancer, heart disease, or emphysema (recorded as: much more/somewhat more/a little more likely and just as likely/less likely).

The perceived benefit of quitting was assessed by asking “How much do you think you would benefit from health and other gains if you were to quit smoking cigarettes permanently in the next 6 months?” (Recorded as: not at all/slightly beneficial and moderately/very much/extremely beneficial).

An overall opinion on smoking cigarettes was assessed by asking: “What is your overall opinion of smoking cigarettes?” (Recorded as: very positive/positive, negative/very negative, and neutral.) Current smokers were also asked whether the following four reasons led them to think about quitting: advice from a health professional, societal disapproval of smoking, close friends’ and family’s disapproval of their smoking, and price of cigarettes (recorded as: not at all and somewhat/very much). For analytic purposes, all responses of “refused” or “don’t know” were excluded.

### 2.3. Statistical Analysis

The data were analyzed using SAS 9.3. Sample characteristics (unweighted) are summarized in Table 1. Smoking-related measures (weighted) are summarized in Table 2. Univariate survey logistic regressions controlling for demographic variables (i.e., smoker type, age, sex, income, education, and marital status) were conducted to test the association between each of the potential quit intention predictors (independent variable) and quit intentions (dependent variable). Then, a multivariate survey logistic regression model was fitted by stepwise selections for all potential predictors, controlling for age, sex, income, education, and marital status. This model shows the overall impact of each predictor on quit intention after adjusting for all the other variables included in the model. All survey logistic regression models incorporated complex survey sample designs and weights for statistical inferences to minimize potential systematic errors caused by unequal selection probabilities and biases.

## 3. Results

Most participants were male (79.8%), aged 30–59 (77.5%), and had achieved a level of education is equivalent to a university degree or higher (79.9%). Most were married or had common law partners (60.2%) and reported a mid- to upper-level household income (94.8%) (Table 1). The distribution across groups was similar for exclusive (50.3%) and poly-use smokers (49.7%) (Table 1).

While most current smokers reported plans to quit smoking (66.4%), only 6.5% had plans to quit within the next month, 28.9% planned to quit between 1 to 6 months, and 30.9% had plans to quit at some point further in the future (Table 2). Most smokers had previous quit attempts (71.6%) and 48.8% reported a neutral opinion on smoking cigarettes. Most smokers perceived that smoking cessation would result in health benefits (71.7%) and more than two-thirds (67.7%) strongly agreed that they regretted having started smoking. Nearly all smokers (92.2%) were worried that smoking may damage their health in the future (Table 2). 

In the multivariate analysis, factors that were significantly associated with quit intentions included age, marital status, heaviness of smoking index (HSI), past quit attempts, regret of having started smoking, belief that smoking had damaged their health, worry that smoking would damage their health in the future, and perceived health benefits of quitting (Table 3). 

Smokers aged 40–59 were less likely to have quit intentions (AOR = 0.52, 95% CI = 0.28–0.97) compared to smokers aged 19–29. Married/common law smokers were more likely to have a quit intention (AOR = 1.56, 95% CI = 1.00–2.44). More strongly nicotine dependent respondents (based on HSI) were less likely have quit intentions (AOR = 0.82, 95% CI = 0.72–0.93). Smokers who had ever tried to quit smoking but did not have a quit attempt in the past year (AOR = 1.99, 95% CI = 1.17–3.36) or had one or more quit attempts in the past year (AOR = 5.93, 95% CI = 3.83–9.18) were more likely to have quit intentions compared to smokers who had never attempted to quit. 

Smokers who regretted having ever started smoking (i.e., strongly agreed/agreed) were significantly more likely to have quit intentions (AOR = 1.83, 95% CI = 1.19–2.83) compared to those who were neutral (i.e., neither agree nor disagree). Those who believed that smoking had damaged their health (AOR 1.71, 95% CI = 1.11–2.63) were more likely to intend to quit compared to those who did not express such a belief. Those who were worried that smoking would damage their health in the future (AOR = 2.70, 95% CI = 1.15–6.36) were more likely to have quit intentions than those who did not worry at all. Smokers who perceived future health benefits to quitting smoking were more likely to have quit intentions (AOR = 1.58, 95% CI = 1.04–2.42) than those who did not perceive health benefits. 

Other factors, including concurrent use of cigarettes with HTPs or NVPs, receiving advice from health professionals, perception of societal disapproval, and the price of cigarettes were not significantly associated with having quit intentions. Concurrent use of NVPs or HTPs was a significant predictor of having higher quit intentions in univariate logistic regression, but no significant difference in quit intentions between exclusive and concurrent users was found in multivariate logistic regression analysis. 

## 4. Discussion

This study shows that most smokers in South Korea have an intention to quit smoking (66.4%) but less than half of them (35.4%) intend to quit within 6 months. This intention to quit rate (within 6 months) in South Korea is lower than those of other high- and middle-income countries like Brazil (48%), Malaysia (44%), Canada (43%), Australia (42%), and the Netherlands (36%), but is higher than those of France (33%), United States (33%), China (28%), Mauritius (28%), Mexico (24%), Japan (11%), Germany (10%), and Kenya (14%) [12,19,30,31]. This study identified eight factors that were significantly associated with quit intentions among adult Korean smokers, namely age, marital status, past quit attempts, nicotine dependence, regretting initiating smoking, reporting that smoking damaged their health, worrying about future health, and perceiving health benefits of quitting smoking (Table 3). 

Sociodemographic factors have been shown to be associated with quit intentions in previous studies [14,16,17,21,25,26,27,32]. However, the first ITC Korea cohort study (2005) on predictors of quit intentions among Korean smokers found that among sociodemographic factors, only educational level and religious affiliation were significant predictors of quit intentions [18]. The association between age and quit intentions has been inconsistent [16,25,26,27]. Our study has shown that other sociodemographic variables, such as age and marital status, are associated with quit intentions. While some studies have shown that older men are more likely to have quit intentions [26], other studies [16,25,27], including our own, show the opposite, where older Korean smokers are less likely to have quit intentions. This may be due to the fact that older smokers have high nicotine dependency, having smoked for a longer period of time, and smoking has become part of their lifestyle, thereby making it less likely for them to have quit intentions [32]. This finding highlights the importance of cessation interventions targeting younger smokers, since quitting becomes more difficult as they grow older [32]. Additionally, smokers with spouses or partners were more likely to have an intention to quit smoking than smokers who were single. Receiving advice from a significant other to quit smoking was previously shown to be related to quit intentions [28]. This finding highlights the significant role of spouses or partners in encouraging smokers to want to quit. 

The association between past quit attempts and the intention to quit in this study corroborates the findings of previous studies in other countries [12,13,15,16,17,21]. Our study is the first to show that past quit attempts were associated with quit intentions among Korean smokers. This result is important because smokers typically need to make multiple quit attempts to increase their chances of success at quitting smoking [33]. Our study suggests that tobacco cessation support programs in South Korea should encourage smokers to continue making quit attempts, even if they fail many of their attempts. In addition, consistent with previous studies, our study shows that smokers who reported high nicotine dependency were less likely to have quit intentions [12,16,20,22,25,26,27]. Nicotine dependence was assessed using HSI, which includes the two components of the Fagerström test that are the most predictive of quitting outcomes: the time to first cigarette and cigarettes per day [34]. Those who are highly dependent on nicotine need to be encouraged to reduce their nicotine dependency in order to help boost future cessation [35]. This can be achieved in many ways, including using nicotine replacement therapies or pharmacotherapies [36] as well as increasing smokers’ tobacco-related knowledge, which was associated with lower nicotine dependency in smokers [24].

Our study found that Korean smokers who regretted ever having started smoking (i.e., strongly agreed/agreed) were significantly more likely to have quit intentions compared to smokers who were neutral. In previous studies, regret was significantly associated with quit intentions in an ITC study of four Asian countries (Thailand, South Korea, Malaysia, and China) [37] and was correlated with quit intentions in another ITC study of four western high-income countries (Canada, US, UK, and Australia) [38]. These studies found that countries that have stronger national tobacco control policies and more negative social norms towards smoking tend to report a higher prevalence of regret among smokers [37,38,39]. Since regret serves as a powerful emotional motivator for future behavior change, anti-smoking interventions and campaigns need to integrate approaches that not only rely on rational explanations, but also appeal to the emotions of smokers to effectively induce quit intentions [39]. 

Consistent with previous studies, our study shows that motivational factors, such as believing that smoking damages health, worrying about future health, and perceiving health benefits of quitting, were significantly associated with quit intentions in smokers [12,15,16,17,21]. This suggests that the motivation for quit intentions among Korean smokers is derived from personal concerns regarding health and quality of life, rather than from external factors like social norms. Given that no other study has previously demonstrated the relative importance of personal beliefs to societal norms in quit intentions among Korean smokers, our novel finding has important implications for tobacco control policy in South Korea, which in the past has mainly emphasized on socially restrictive measures, such as smoking bans in public places. Our findings suggest that more focus should be given to educational communication campaigns that highlight how tobacco use personally affects smokers’ health and quality of life. Although strong tobacco control regulations focusing on increasing social norms on smokers have managed to decrease smoking prevalence to a certain extent in South Korea, smoking rates have recently reached a plateau [5,8]. This phenomenon may be attributed to resistance based on intrinsic health-related knowledge and attitudes regarding tobacco use that were not sufficiently modified by social policies [40]. Thus, individual-based interventions addressing these deep-seated beliefs and knowledge on the effects of tobacco use on personal health and well-being may be necessary to further bring down smoking prevalence rates in South Korea. Additionally, tobacco control policies, including increasing tobacco prices and expanding outdoor smoking bans, should be strengthened as well to increase the strength of social norms to motivate Korean smokers to quit smoking.

Despite the rise of HTP and NVP use in recent years, we found no evidence that concurrent Korean smokers using HTPs and/or NVPs were more likely to have quit intentions than exclusive smokers. The proportions of South Korean users of HTPs and NVPs are rising steadily [41,42]. According to the Korea National Health and Nutrition Examination Survey (KNHANES), the prevalence of current e-cigarette use among adults increased from 1.1% (male 2.0%, female 0.3%) in 2013 to 3.2% (male 5.2%, female 1.1%) in 2020 [43]. According to the Community Health Survey data, the prevalence of current HTP use among Korean men also increased from 4.5% in 2018 to 6.7% in 2019 (median value) [44]. 

Moreover, most users of NVPs or HTPs also tend to concurrently smoke cigarettes [45,46]. Over 80% of current HTP users in South Korea were either concurrent users with either cigarettes or e-cigarettes (ECs) or triple users [45]. Likewise, over 80% of NVP users were concurrent users with cigarettes [46]. HTPs and NVPs are often marketed as less harmful products compared to cigarettes, and users of these products are perceived to be able to have higher quit intentions and quit more easily than cigarette smokers [47,48]. However, few studies have explored the role of concurrent use in determining quit intentions. Our study provides evidence that the concurrent use of cigarettes with an alternative nicotine product was not significantly associated with quit intentions (AOR = 1.22, 95% CI = 0.81–1.82). While univariate logistic regression analysis suggested a significant association between concurrent use and quit intentions, this relationship was attenuated with multivariate logistic regression. This novel finding of the lack of association between concurrent use and quit intentions has not been demonstrated elsewhere. 

While most of the current regulations on combustible cigarettes also apply to NVPs and HTPs, especially those concerning taxation, additional measures are needed, such as those enforcing companies to be more transparent about the ingredients contained in these products [49]. Further measures should include central regulatory agencies conducting more independent research on the health hazards of NVPs/HTPs as well as effectively conveying this information to the public [49]. Additionally, policymakers should tailor regulations to fit the unique characteristics of the NTP/HTP market, such as the prevalence of illegal online sales [49]. Furthermore, given the rising rates of concurrent smokers, future studies should not only investigate the association of concurrent smoking with quit intentions, but also with actual quit success.

The strength of this study includes the use of population-based data and the inclusion of known standardized measures associated with quit intentions, which have been used in previous studies of other countries participating in the ITC Project. The limitations of our study include the use of self-reporting, which may result in recall bias and social desirability. 

## 5. Conclusions

This study shows that most Korean smokers are interested in quitting (66.4%) and predictors of quit intentions are consistent with those of many other countries (e.g., Canada, US, UK, Australia, China, Malaysia, Mauritius, and Kenya). Determinants of quit intentions included sociodemographic variables (i.e., age and marital status), HSI, past quit attempts, regretting having started smoking, believing that smoking lowered quality of life and damaged their health, worrying that smoking would damage health in the future, and perceiving health benefits of quitting. Our study contributes to the current understanding of quit intentions among South Korean adult smokers by showing that intrinsic health-related beliefs tend to be more important than societal norms in shaping quit intentions among Korean smokers, and that the concurrent use of cigarettes with HTPs or NVPs is not significantly associated with quit intentions. These novel findings should be considered in shaping future smoking cessation policies, such as reinforcing smoking cessation and education programs that focus on shaping personal beliefs, encouraging multiple quit attempts, lowering nicotine dependence, and including measures to discourage concurrent tobacco or nicotine product use in South Korea.

## Figures and Tables

**Table 1:** Sample characteristics among Korean adult smokers (unweighted).

	N	%
**Sex**		
Male	3013	79.8
Female	765	20.2
**Age**		
19–29	513	13.6
30–39	1032	27.3
40–59	1895	50.2
60+	338	8.9
**Education (derived)**		
Less than middle school	32	0.9
High school graduate or some university	723	19.2
University degree or higher	3011	79.9
**Marital status**		
Single	1345	35.9
Married/common law	2263	60.2
Separated/divorced/widowed	151	0.9
**Household income (derived)**		
Low income (<10 m KRW)	124	3.3
Middle income (10 m to 60 m KRW)	1930	51.1
Upper income (>60 m KRW)	1651	43.7
Not stated (refused/don’t know)	73	1.9
**Smoking Type**		
Exclusive smokers	1900	50.3
Concurrent smokers	1878	49.7

**Note**: Respondents who answered “refused” or “don’t know” were excluded from the analyses. Respondent answers of “refused” or “don’t know” to the income question were considered valid.

**Table 2:** Smoking-related measures (weighted).

Measures	N	%	95% CI
**Intention to quit**			
No plan to quit	916	33.7	30.7–36.5
Plan to quit	2527	66.4	63.5–69.2
**Time plan to quit smoking**			
Within the next month	245	6.5	5.1–8.0
Between 1 to 6 months from now	1033	28.9	26.2–31.6
Sometime in the future, beyond 6 months	1249	30.9	28.2–33.6
Not planning to quit	916	33.6	30.8–36.5
**Ever tried to quit smoking**			
Yes	2760	71.6	69.0–74.2
No	975	28.4	25.8–31.0
**Overall opinion of smoking cigarettes**			
Negative/very negative	1491	40.1	37.3–42.9
Neutral	1739	48.8	46.0–51.7
Very positive/positive	451	11.03	9.3–12.8
**Perceived health benefit of quitting**			
Moderately/very much/extremely	2553	71.7	69.1–74.3
Not at all/slightly	1033	28.3	25.7–30.9
**Regret ever starting smoking**			
Strongly agree/agree	2491	67.7	65.0–70.4
Neither agree nor disagree	786	20.3	18.0–22.6
Strongly disagree/disagree	386	12.0	10.1–13.9
**Smoking lowered the quality of life**			
A fair amount/a great deal	1018	28.5	25.7–31.2
Not at all/just a little	2306	71.5	68.8–74.3
**Worried that smoking will damage future health**			
Not at all worried	195	7.8	6.2–9.4
A little/moderately worried/very worried	3418	92.2	90.6–93.8

**Note**: Respondents who answered ”refused” or “don’t know” were excluded case-wise from the analyses.

**Table 3:** Factors associated with having an intention to quit smoking among Korean adult smokers.

Predictor	Category	Multivariable Model AOR (95% CI)
Sex	Male	Ref
	Female	0.74 (0.37–1.49)
Age	19–29	Ref
	30–39	0.60 (0.32–1.12)
	40–59	0.52 (0.28–0.97) *
	60+	0.42 (0.17–1.04)
Education	Less than middle school graduate	Ref
	High school graduate/ some university	2.35 (0.59–9.38)
	University degree or higher	3.26 (0.82–13.02)
Marital status	Single	Ref
	Married/common law	1.56 (1.00–2.44) *
	Separated/divorced/widowed	1.89 (0.78–4.58)
Household income	Low income (<10 m KRW)	Ref
	Middle income (10 m to 60 m KRW)	1.20 (0.56–2.54)
	Upper income (>60 m KRW)	0.97 (0.42–2.20)
	Not stated	0.93 (0.21–4.15)
Tobacco use status	Exclusive smoker	Ref
	Concurrent smoker	1.22 (0.81–1.82)
Heaviness of Smoking Index		0.82 (0.72–0.93) *
Quit attempt	Never tried to quit	Ref
	No quit attempt in the past year	1.99 (1.17–3.36) *
	One or more past quit attempts in past year	5.93 (3.83–9.18) *
Regret ever starting smoking	Neither agree nor disagree	Ref
	Strongly agree/agree	1.83 (1.19–2.83) *
	Strongly disagree/disagree	0.91 (0.44–1.87)
Smoking is important to life	Strongly agree/agree	Ref
	Neither agree nor disagree	1.48 (0.98–2.24)
	Strongly disagree/disagree	1.62 (0.93–2.83)
Smoking lowered quality of life	Not at all/just a little	Ref
	A fair amount/a great deal	1.03 (0.66–1.61)
Smoking damaged health	Not at all/just a little	Ref
	A fair amount/a great deal	1.71 (1.11–2.63) *
Worried smoking will damage future health	Not at all A little/moderately/very much	Ref 2.70 (1.15–6.36) *
Likelihood of getting a smoking-related diseases	Much more/somewhat more/ a little More likely	Ref
	Just as likely/less likely	0.68 (0.39–1.18)
Health benefits of quitting	Not at all/slightly Moderately/very much/extremely	Ref 1.58 (1.04–2.42) *
Overall opinion of smoking cigarettes	Very positive/positive Neutral Very negative/negative	Ref 0.69 (0.37–1.30) 1.21 (0.60–2.45)
Reason that led to thinking about quitting: advice from a health professional	Not at all	Ref
	Somewhat/very much	1.44 (0.99–2.11)
Reason that led to thinking about quitting: society disapproves smoking	Not at all	Ref
	Somewhat/very much	1.37 (0.87–2.17)
Reason that led to thinking about quitting: close friends and family disapprove	Not at all Somewhat/very much	Ref 1.40 (0.91–2.15)
Reason that led to thinking about quitting: price of cigarettes	Not at all	Ref
	Somewhat/very much	1.37 (0.86–2.10)

**Note**: Respondents who answered “refused” or “don’t know” were excluded from the analyses. *: statistically significant (*p* value < 0.05); Heaviness of Smoking Index, mean (SD) = 2.03 (0.04).

## Data Availability

In each country participating in the International Tobacco Control Policy Evaluation (ITC) Project, the data are jointly owned by the lead researcher(s) in that country and the ITC Project at the University of Waterloo. Data from the ITC Project are available to approved researchers 2 years after the date of issuance of cleaned data sets by the ITC Data Management Centre. Researchers interested in using ITC data are required to apply for approval by submitting an International Tobacco Control Data Repository (ITCDR) request application and subsequently signing an ITCDR Data Usage Agreement. The criteria for data usage approval and the contents of the Data Usage Agreement are described online (http://www.itcproject.org) (accessed on 25 February 2022).

## Supplementary Materials

The following supporting information can be downloaded at: https://www.mdpi.com/article/10.3390/ijerph191710839/s1, Table S1: Summary of related literature on factors associated with quit intentions.
