# Supplementary material for: Factors Associated with Quit Intentions among Adult Smokers in South Korea: Findings from the 2020 ITC Korea Survey
Source: Int J Environ Res Public Health. 2022 Aug 31;19(17):10839. doi: 10.3390/ijerph191710839 (PMC9518068; doi:10.3390/ijerph191710839)
Supplement: Supplementary file 1 [file ijerph-19-10839-s001.zip › ijerph-1854133-supplementary.pdf]

Supplementary Table S1. Summary of related literature on factors associated with quit intentions.

Reference	Objective	Finding	Limitation
**Korea**			
Myung et al. 2012 [1]	Using ITC survey data from 2005, the association between sociodemographic factors, smoking-related beliefs, and smoking restrictions with quit intentions among Korean adults was assessed.	In multiple logistic regression analysis, higher education level, having a religious affiliation, and a higher self-efficacy regarding quitting were significantly associated with quit intentions.	The cross-sectional nature of the study prevents inference of causal effect. Small number of adult female smokers.
Park et al. 2014 [2]	The 2010 Community Health Survey data was used to determine factors associated with quit intentions among Korean women smokers.	In logistic regression analysis, age, health-related quality of life, perceived stress, marital status, age of starting to smoke, number of cigarettes per day, exposure to smoking-quitting campaigns, previous attempts at weight control, frequency of alcohol use, hypercholesterolemia, past quit attempts, and regular medical check-ups were associated with the intention to quit.	Cross-sectional nature of the data limits inference of causal effect. Limitations considered regarding variables used, i.e., since this study is a secondary analysis, there were limitations in selecting and constructing variables that affect quit intentions.
Ahn et al. 2015 [3]	The 2013 Community Health Survey data was used to determine factors associated with quit intentions in community-dwellings among male adult smokers in Korea.	In logistic regression analysis, age, amount of smoking per day, previous attempts to quit, exposure to smoking cessation campaigns, and brushing teeth after lunch were significantly associated with the quit intentions.	Cross- nature of the data limits inference of causal effect. A limitation is considered regarding the variables used since this study is a secondary analysis. Sample from a single province and may not be representative of the entire population.
Kim et al. 2017 [4]	The 6th KNHANES (2013-2014) survey data was used to determine the factors associated with quit intentions among Korean adult males.	In multiple logistic regression analysis, the intention to quit was associated with old age, fewer packs smoked per day, and having chronic diseases.	Cross-sectional nature of the study limits inference of causal effect.
Choi et al. 2018 [5]	Using ITC survey data from 2015-2016, the association between HSI and quit intentions was assessed.	In multivariate logistic regression analysis, smokers with higher HSI or no quit intentions tended to have lower knowledge regarding the health risks of smoking.	Cross-sectional study prevents inference of causal effect. Study only included smokers, and thus comparisons between nonsmokers and smokers could not be made. The effect of recent updates to pictorial warnings on packaging concerning lung and oropharyngeal cancer risk could not be evaluated.
Lee et al.	Using the 5th and 6th	In multiple logistic regression analysis, quit intention was	Cross-sectional study prevents inference of causal effect. A

Reference	Objective	Finding	Limitation
2020 [6]	KNHANES survey data from 2010-2015, the factors associated with quit intentions in elderly Korean men was assessed.	significantly associated with history of ischemic heart disease.	limitation is considered regarding the variables used since this study is a secondary analysis. Small sample size of study participants. Recall bias due to self-reported questionnaires.
Hwang et al. 2021 [7]	The 2017 Community Health Survey data was used to determine whether smoking cessation intentions are associated with advice to quit from significant others and medical professionals.	In multinomial multiple logistic regression analysis, smokers advised to quit by both significant others and medical professionals, by significant others only, and by medical professionals only were more likely to intend to quit within 1 month than those not advised to quit.	The cross-sectional nature of the data limits inference of causal effect. The identity of the significant other was not described. The sample was from a single province and may not be representative of the entire population.
**International**			
Siahpush et al. 2004 [8]	Using Wave 1 (2002) of the ITC Four Country Survey, the effect of SES on nicotine dependence, self-efficacy, and intentions to quit was assessed among adult smokers in the U.S., Canada, U.K., and Australia.	In logistic regression analysis, those with low education had 40% larger odds of having no quit intentions than those with high education. Those with low income had 23% larger odds of having no quit intentions than those with high income. Lower SES smokers tend to be more addicted and are likely to need more support in smoking cessation.	Analysis is limited to daily smokers.
Yu et al. 2004 [9]	A cross sectional study of patients attending medical and surgical Specialist Outpatient Clinic in Hong Kong from October 2000 to June 2001 was conducted to determine factors associated with the intention to quit among adult smokers in Hong	In stepwise forward logistic regression analysis, past quit attempts, receiving information from sources other than doctors, believing that doctor's advice was useful, believing that all smokers should quit smoking, and a positive attitude towards quitting were significantly associated with quit intentions.	Using self-reported data may introduce bias. Small sample size may have limited the significance of some variables. Conducting multiple significance tests may have increased the likelihood of type 1 error.

Reference	Objective	Finding	Limitation
	Kong.		
Abdullah et al., 2005 [10]	To assess the prevalence of each step in the smoking-cessation process (intentions to quit, attempts to quit, and successful quitting) and to examine the factors associated with them among Chinese smokers.	Being male, married, and not smoking to kill time were associated with intentions to quit smoking	The study may not be wholly representative, since occasional smokers were excluded from study. Secondly, the question on ''whether the smokers want to quit smoking'' was only asked of those who had never tried to quit smoking. It was assumed that those who had tried in the past to quit smoking already intended to quit smoking. Thus, the association of factors with quit intentions only referred to past intentions to quit instead of quitting intentions at the time of enumeration. As a result, the number of previous attempts to quit, which was a significant factor in predicting intention to quit in various studies could not be a predictor in their analysis.
Fagan et al. 2007[11]	Using data from the national 2003 Tobacco Use Supplement to the Current Population Survey, factors associated with quit attempts and the intentions to quit among young American adult smokers aged 18-30 years were assessed.	In multivariate logistic regression analysis, nicotine dependence measures were significantly associated with quitting and quit intentions among daily smokers; sociodemographic variables were associated with quitting and quit intentions among nondaily smokers.	Cross-sectional study design limits determining causality. Small sample size for certain subgroups limits power of analyses.
Feng et al. 2010 [12]	Using wave 1 ITC survey data from 2006, factors associated with the quit intentions among Chinese adult smokers were assessed.	In multivariate logistic regression analysis, past quit attempts, duration of past attempts, HSI, outcome expectancy of quitting, worry about future health, and overall opinion of smoking were significantly associated with quit intentions.	The use of self-reported data may introduce recall bias and social desirability bias. Survey response rates in the first wave were moderate to low. SES variables were difficult to measure and certain measures had a large proportion of "unknown" responses (ex. income). Survey limited to urban areas inhabited mainly by Han Chinese.
Driezen et al. 2016 [13]	Using Wave 2 of the ITC Bangladesh Survey, factors associated with the quit intentions among adult smokers in Bangladesh were assessed.	In stepwise backward logistic regression analysis, area of residence, number of cigarettes smoked daily, previous quit attempt, visiting a doctor in the past, having a child aged 5 or below at home, perceived benefit from quitting, being worried about own health, knowledge of SHS, not enjoying smoking, and workplace smoking policy were significantly associated with quit intentions.	Findings were limited to daily smokers. Nicotine dependence could not be included as a predictor of quit intentions due to the way in which data was collected.

Reference	Objective	Finding	Limitation
Kaai et al. 2016 [14]	Using Wave 1 (2009) of the ITC Mauritius Survey, factors associated with the quit intentions among adult smokers in Mauritius were assessed.	In multivariate logistic regression analysis, longer duration of past quit attempts, perceiving benefits of quitting, worrying about smoking damaging health in the future, and not enjoying smoking were significantly associated with quit intentions.	Using self-reported data may introduce recall bias and social desirability bias. Cross-sectional study design limits determining causality. Small female sample size.
Kaai et al. 2019 [15]	Using 2012 ITC Kenya and Zambia Surveys, factors associated with quit intentions among smokers in two African countries were assessed.	In multivariate logistic regression analysis, younger age, previous quit attempts, perceiving that quitting is beneficial to health, worrying about future health consequences of smoking, and low nicotine dependence were significantly associated with quit intentions in both countries. Additionally, in Zambia only, having a quit attempt lasting more than 6 months, lower smoking enjoyment, having negative opinion on smoking, and concern about expenses were significant predictors of quit intentions.	Using self-reported data may introduce recall bias and social desirability bias. Cross-sectional study design limits determining causality.
Hasan et al. 2022 [16]	Using 2020 ITC Malaysia survey data, factors associated with quit intentions among Malaysian adult smokers were assessed.	In multivariate logistic regression analysis, Malay ethnicity, higher level of education, previous quit attempts, receiving advice to quit from a healthcare provider, and worrying about future health were significantly associated with quit intentions.	The use of self-reported data may introduce recall bias and social desirability bias. Cross-sectional study design limits determining causality. Factors like depression and self-efficacy were not measured in the study.

References

1. Myung, S.K., et al., *Association of sociodemographic factors, smoking-related beliefs, and smoking restrictions with intention to quit smoking in Korean adults: findings from the ITC Korea Survey.* J Epidemiol, 2012. **22**(1): p. 21-7.
2. Park, K.Y., *Predictors of Intention to Quit Smoking among Woman Smokers in Korea.* Journal of Korean Academy of Fundamentals of Nursing, 2014. **21**(3): p. 253-263.
3. Ahn, H.-R., *Factors Associated with Intention to Quit Smoking in Community-dwelling Male Adult Smokers.* jkachn, 2015. **26**(4): p. 364-371.
4. Kim, S.-J., et al., *Factors Associated with Intention to Quit Smoking in Korean Adult Males: The Sixth Korean National Health and Nutrition Examination Survey, 2013-2014.* Korean Journal of Family Practice, 2017. **7**(2): p. 276-280.
5. Choi, Y.-J., et al., *Tobacco Related Knowledge Is Associated with Heaviness of Smoking Index (HSI) and Intention to Quit among Korean Smokers: Findings from 2016 ITC Korea Survey.* Journal of the Korean Society for Research on Nicotine and Tobacco, 2018. **9**(0): p. 31-38.
6. Lee, I., et al., *Factors Associated with the Intention to Quit Smoking in Elderly Korean Men: The Korea National Health and Nutrition Examination Survey 2010-2015.* Korean J Fam Med, 2020. **41**(4): p. 237-242.
7. Hwang, J.H. and S.-W. Park, *Smoking Cessation Intention and Its Association with Advice to Quit from Significant Others and Medical Professionals.* International Journal of Environmental Research and Public Health, 2021. **18**(6): p. 2899.

8. Siahpush, M., et al., *Socioeconomic variations in nicotine dependence, self-efficacy, and intention to quit across four countries: findings from the International Tobacco Control (ITC) Four Country Survey.* Tob Control, 2006. **15 Suppl 3**(Suppl 3): p. iii71-5.
9. Yu, D.K., et al., *Smoking cessation among Hong Kong Chinese smokers attending hospital as outpatients: impact of doctors' advice, successful quitting and intention to quit.* Asia Pac J Public Health, 2004. **16**(2): p. 115-20.
10. Abdullah, A.S.; Yam, H.K. Intention to quit smoking, attempts to quit, and successful quitting among Hong Kong Chinese smokers: population prevalence and predictors. *Am J Health Promot* **2005**, *19*, 346-354, doi:10.4278/0890-1171-19.5.346.
11. Fagan, P., et al., *Quit attempts and intention to quit cigarette smoking among young adults in the United States.* Am J Public Health, 2007. **97**(8): p. 1412-20.
12. Feng, G., et al., *Individual-level factors associated with intentions to quit smoking among adult smokers in six cities of China: findings from the ITC China Survey.* Tob Control, 2010. **19 Suppl 2**(Suppl_2): p. i6-11.
13. Driezen, P., et al., *Determinants of intentions to quit smoking among adult smokers in Bangladesh: findings from the International Tobacco Control (ITC) Bangladesh wave 2 survey.* Glob Health Res Policy, 2016. **1**: p. 11.
14. Kaai, S.C., et al., *Predictors of quit intentions among adult smokers in Mauritius: Findings from the ITC Mauritius Survey.* Tobacco Prevention & Cessation, 2016. **2**(75).
15. Kaai, S.C., et al., *Identifying factors associated with quit intentions among smokers from two nationally representative samples in Africa: Findings from the ITC Kenya and Zambia Surveys.* Prev Med Rep, 2019. **15**: p. 100951.
16. Hasan, S.I., et al., *Who Are More Likely to Have Quit Intentions among Malaysian Adult Smokers? Findings from the 2020 ITC Malaysia Survey.* Int J Environ Res Public Health, 2022. **19**(5).
